# Evidence Integration in Natural Acoustic Textures during Active and Passive Listening

**DOI:** 10.1523/ENEURO.0090-18.2018

**Published:** 2018-04-13

**Authors:** Urszula Górska, Andre Rupp, Yves Boubenec, Tansu Celikel, Bernhard Englitz

**Affiliations:** 1 Department of Neurophysiology, Donders Institute, Radboud University Nijmegen, The Netherlands; 2 Psychophysiology Laboratory, Institute of Psychology, Jagiellonian University, Krakow, Poland; 3 Smoluchowski Institute of Physics, Jagiellonian University, Krakow, Poland; 4 Section of Biomagnetism, Department of Neurology, University of Heidelberg, Heidelberg, Germany; 5 Laboratoire des Systèmes Perceptifs, CNRS UMR 8248, Paris, France; 6 Département d’Études Cognitives, École Normale Supérieure, PSL Research University, Paris, France

**Keywords:** decision making, EEG, evidence integration, natural stimuli, stimulus statistics

## Abstract

Many natural sounds can be well described on a statistical level, for example, wind, rain, or applause. Even though the spectro-temporal profile of these acoustic textures is highly dynamic, changes in their statistics are indicative of relevant changes in the environment. Here, we investigated the neural representation of change detection in natural textures in humans, and specifically addressed whether active task engagement is required for the neural representation of this change in statistics. Subjects listened to natural textures whose spectro-temporal statistics were modified at variable times by a variable amount. Subjects were instructed to either report the detection of changes (active) or to passively listen to the stimuli. A subset of passive subjects had performed the active task before (passive-aware vs passive-naive). Psychophysically, longer exposure to pre-change statistics was correlated with faster reaction times and better discrimination performance. EEG recordings revealed that the build-up rate and size of parieto-occipital (PO) potentials reflected change size and change time. Reduced effects were observed in the passive conditions. While P2 responses were comparable across conditions, slope and height of PO potentials scaled with task involvement. Neural source localization identified a parietal source as the main contributor of change-specific potentials, in addition to more limited contributions from auditory and frontal sources. In summary, the detection of statistical changes in natural acoustic textures is predominantly reflected in parietal locations both on the skull and source level. The scaling in magnitude across different levels of task involvement suggests a context-dependent degree of evidence integration.

## Significance Statement

The everyday auditory environment is often complex and highly variable. Separating relevant changes from irrelevant variability is important for auditory processing. Previous research has already shown that sensory evidence is integrated to achieve accurate decisions. In the present study, we extend this research in two ways, first by using more realistic and complex stimuli, and second, by modulating the level of engagement of subjects in the task. We demonstrate that dynamic processing of natural stimuli leads to similar neural responses as for synthetic complex stimuli. Further, neural responses continue to reflect properties of the acoustic stimulus even for reduced task engagement, albeit with much lower amplitudes. We conclude that auditory evidence integration may continue even during passive perception.

## Introduction

Changes in the sensory environment provide important information to avoid danger or realize opportunities. In an ever-changing environment, humans need to selectively react to informative changes and ignore others. For example, a single drop of water in silence may indicate a dripping faucet, but should be ignored while it is raining. Hence, the importance of a stimulus can only be determined if its surrounding context is known, i.e., the properties of the preceding stimuli and the predictions it allows. Generally, if a stimulus is predictable from the past, it can be directly taken into account in the behavioral planning, while unpredictable stimuli may catch one off-guard.

Many natural stimuli can be described on a statistical level, e.g., the temporal distribution of raindrops. These stimuli are referred to as textures both in vision (Julesz, 1962, 1980; Ziemba et al., 2016) and audition (Kelly and O’Connell, 2013; Boubenec et al., 2016). In an auditory texture, a listener needs to estimate the acoustic statistics of the context, to separate predictable from unpredictable changes (Piazza et al., 2013; Kaya and Elhilali, 2014; Simpson et al., 2014). As shown previously, human listeners can make use of statistical information for the recognition of sounds (McDermott et al., 2013). Further, it has been demonstrated that textural sounds can be recreated from samples using only their statistics (McDermott and Simoncelli, 2011), which remain recognizable by human listeners. So far, the neural basis for the representation of these statistics has only been partially addressed. Subcortical stations have been demonstrated to be sensitive to a limited set of stimulus statistics (Simpson et al., 2013; Malmierca et al., 2015) while longer stimulus timescales are correlated with activity in higher-order cortical areas (Overath et al., 2008). Furthermore, the detection of statistical changes in auditory textures correlates with increased neuronal responses in auditory association cortex (Overath et al., 2010). Recently, it was suggested that evidence integration in change detection tasks (Kelly and O’Connell, 2013; Boubenec et al., 2017) is linked to the formation of a centro-parietal positivity (CPP) potential in EEG recordings. In complex and in particular in statistically defined stimuli, the ability to detect a change, by definition, requires the estimation of stimulus properties over a reference period, and concurrently accumulating sensory evidence for a potential change in statistics. Recently, we suggested dynamic statistical models for this dual-estimation task, which mimic human performance for unexpected changes (Boubenec et al., 2017).

In the present study, we investigated the neural basis of sensory evidence accumulation in a dynamic, naturalistic stimulus condition, and compared it across the different levels of task involvement, active and two passive conditions (see last paragraph). The task design is chosen to mimic real-world challenges, where complex changes occur at an unexpected time in an unattended stimulus, e.g., an environmental sound. Whole-brain responses are recorded using scalp EEG and processed using detailed source-analysis to determine the underlying neural generators. Our main hypothesis is that change detection in complex, statistically defined, textural stimuli requires the accumulation of sensory evidence, and that the degree to which the neural system performs the accumulation covaries with the level of task involvement.

We find the properties of the change in statistics to be represented most clearly in parietal sources; consistent with other decision-making tasks in the visual (Shadlen and Newsome, 2001; Kelly and O’Connell, 2013) and auditory (O’Connell et al., 2012; Kelly and O’Connell, 2013) domain. In the present data, a change in stimulus statistics evokes an ERP (event related potential) in parietal cortex. Its rising slope and size scale with the size of the change in statistics (“mixing coefficient”) and with the exposure duration (“change time”), i.e., with the quality of the estimation of the statistics before the change. Consistently, source activations in both parietal and auditory regions scale their amplitudes, while they remain unchanged for frontal cortex.

We observed that the parietal responses scale with the subjects' acquaintance with the task: they were larger in active than in the passive groups (passive-aware and passive-naive), while the P2-like responses from auditory cortex remain very similar. This suggests that the ability to track changes in sound statistics remains partially available during nonactive listening, similar to previous experiments where unattended sources of information were still neurally represented, e.g., in dichotic listening tasks (Ding and Simon, 2012; Schepman et al., 2015). Hence, the degree of sensory evidence accumulation about regularities in the acoustic environment varies according to task involvement.

## Materials and Methods

All experiments were performed in accordance with the directives of the Helsinki Declaration (1975, revised 2000) and approved by the Ethical Committee of the Institute of Psychology, Jagiellonian University. Before the procedure started, all participants signed a written consent form.

### Participants

The corresponding groups of subjects took part in the two phases of the natural auditory texture experiment. In the active variant 18 volunteers (eight female, age: 30.6 ± 10.1 years, mean ± SEM, higher education, at least undergraduate) participated and in the passive paradigm we collected data from 18 subjects (nine female, age: 29.7 ± 10.6 years, higher education, at least undergraduate). A subgroup of eight subjects participated in both paradigms, first in the active and then in the passive. The passive subgroups will be treated separately, denoted passive-aware (first active, then passive) and passive-naive (passive only). Only one subject from the active group was excluded, due to excessive noise in the EEG, such that *N* = 17 subjects entered the active EEG analysis. All participants reported normal hearing and no neurologic or psychological disorders and absence of drug abuse and medication.

### Stimulus design

#### Base textures

A specific set of natural texture sounds was prepared for the present study. We used the MATLAB toolbox provided by (McDermott and Simoncelli, 2011) to create different acoustic textures based on real sounds. Briefly, they were constructed by an iterative procedure which modifies a random noise signal until its statistics match the ones of the real signal (for details, see McDermott and Simoncelli, 2011). We chose the textures based on the sound of rain, applause, and bubbles in water, as they fulfilled the criteria of being spectrally and temporally broad and dense (compared to, e.g., random clock ticks or bandpass filtered sounds), and they were perceptually reasonably similar, thus distinguishing them was not trivial. We created multiple samples of each sound which agreed in statistics but differed in spectro-temporal detail. In this way, a sound with a given statistic is not recognizable by a particular realization of the fine-structure. As is common in natural sounds, the base-textures exhibited nonidentical spectra, however, for low mixing-coefficients (see next section) the spectra are nearly matched at the moment of change.

#### Change in statistics

**Figure 1.**
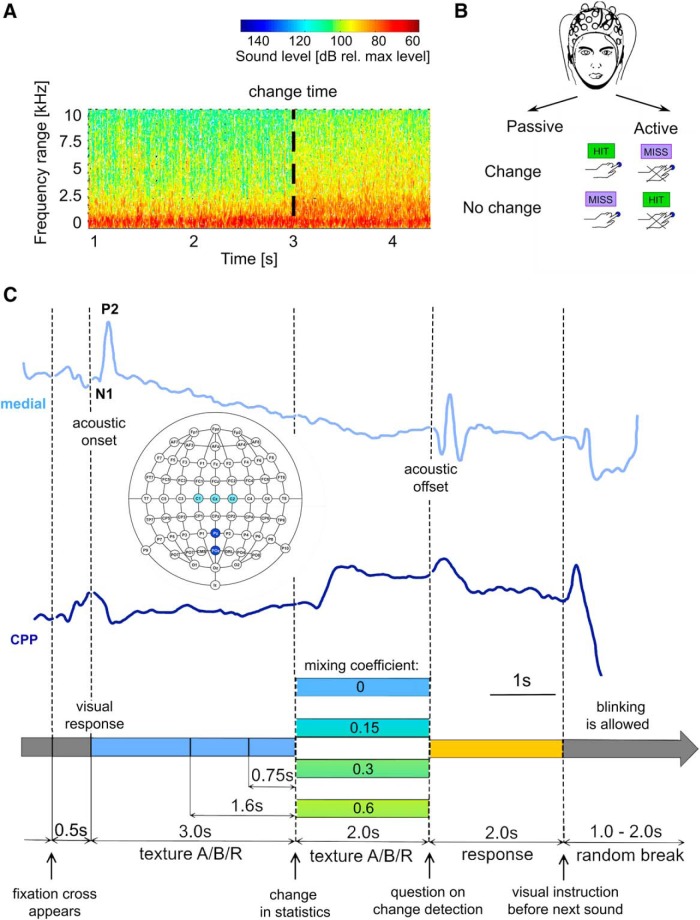
Experimental paradigm for the detection of changed statistics of natural acoustic textures. ***A***, We presented natural textures which change their statistics at random times to a variable degree (depicted: 3 s). The change in statistics leads to a distributed change in spectro-temporal properties. The present example shows a transition from bubbling to a linear mixture between bubbling and rain, with an intermediate mixing coefficient of 0.3. Level is relative to the maximal overall level. ***B***, Texture sounds were provided via headphones while simultaneously recording whole-head EEG signals from 64 channels (10–20 system, see inset in ***C***). In the active paradigm, listeners were instructed to report whether they heard a change by pressing a button after the termination of the sound and otherwise not to respond. Instead, in the passive variant they were just asked to listen to the stimuli. ***C***, Basic design of the change detection paradigm. The trial was started by the appearance of a fixation cross in the center of the screen (prompting subjects to refrain from blinking), followed by the first texture stimulus. After a duration randomly drawn from the three possible change times, the sound continued uninterrupted and at the same level, but with changed statistics (middle, different colors indicate the linear mixing coefficients between the first and the second texture). The choice of first and second texture was always randomized, i.e., it could also stay the same (25% = catch trials). 2 s after the second statistic was presented, the sound was terminated, and the subjects responded (change) or remained silent (no change) within 2 s. The potentials on top show samples from the central (light blue, location on the scalp shown in inset) and the PO/posterior (dark blue) location. The central data shows a prominent N1/P2 complex after stimulus onset, a sustained suppression due to the continuous sound presentation, followed by barely a response after the change, and a final, clear stimulus offset. Conversely, the PO potential shows a response to the fixation cross, but a large response after the change in statistics.

The statistics of the texture stimuli could change during its presentation or stay the same (catch trials; for illustration, see Fig. 1). Changes consisted of a linear mixing between two acoustic textures (Fig. 1*A* shows the spectrogram of the stimulus with a transition at 3 s, computed as the short-term Fourier transform of the acoustic stimulus; more details in the section below). Since we applied 3 base textures, we could create 6 transitions with a change in texture (e.g., rain => applause), and 3 without a change (e.g., rain => rain), which represent catch trials. The latter were also crossfaded, which, however, leaves them statistically unchanged. The time of change was drawn randomly from a set of three time points, i.e., 0.75, 1.6, and 3 s, unbeknownst to the subjects. Catch trials were matched in overall length, to prevent any bias on trial length. The transition between the statistics was implemented as a linear mixing between the two sounds with sigmoidal temporal profile, i.e.,

(1) $$S(t)={W_{1}(t)T}_{1}(t)+W_{2}(t)T_{2}(t)$$

where T_i_(t) are the first and the second texture stimulus (as a sound pressure wave form) and the weighting functions W_i_(t) are given as

(2) $$W_{2}(t)=MC/(1+exp(-(t-CT)/\tau ))$$

i.e., a sigmoidal with a time constant τ = 10 ms centered around the change time CT. MC is the mixing coefficient, which determines the change size in statistics, and thus the difficulty of the task. For noncatch trials, we used three values for MC, i.e., 0.15, 0.3, and 0.6. W_1_(t) is simply given by

$$W_{1}(t)=1-W_{2}(t)$$

Finally, we ensured that the stimulus level did not change through the statistics transition, by defining the final stimulus S_f_(t) to be

$$S_{f}(t)=C(t)S(t)$$

where C(t) is given by

(3) C(t)=1+(std(S(t<CT))/std(S(t>CT))-1)/(1+exp(t-CT)/τ)

which is again a sigmoidal function of time with the same parameters as above. The overall level of the stimulus was adjusted to 70-dB SPL using a sound level meter measured at the output of the speaker (Brüel & Kjær).

To prevent recognition of individual samples, we created statistically matched samples, each of which occurred only once. Ten samples were created for each condition and catch trials were presented twice as often, thus leading to the total number of 720 trials (i.e., six transitions × three change times × three mixing coefficients × 10 samples = 540 signal trials, and three transitions × three change times × 10 samples × two repetitions = 180 trials).

### Experimental procedure

Subjects were seated in an air-conditioned soundproof chamber facing a monitor. The procedure was explained with a standard text, and a short training preceded the experiment. Then the EEG electrodes were attached, followed by a 2-min resting state registration while subjects were instructed to fixate on a white cross displayed in the middle of the screen 0.5 s before the sound. Subjects performed the active and passive conditions on different days.

**Figure 2.**
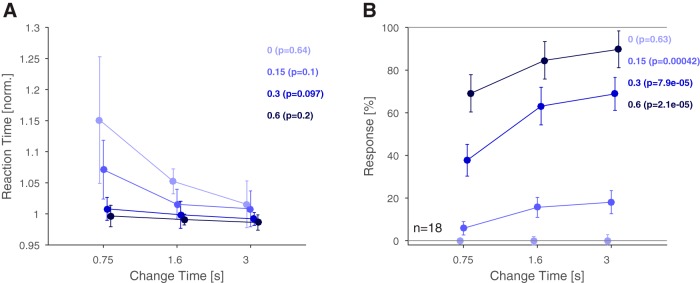
Reaction times and response rates suggest improved performance for stimulus exposure and larger changes in stimulus statistics. ***A***, Reaction times decreased significantly as a function of change time and change size. Within each change size, reaction times did not reach statistical significance for individual change sizes. Reaction times decreased significantly as a function of change size (mixing coefficient, across colours). ***B***, Response rates increased significantly with change time for all change sizes >0. As expected, the response rates did not increase in catch trials (light blue) with change time, indicating that subjects closely listened for changes, rather than responding as a function of time within the trial. Response rates also increased significantly as a function of change size (different colors). In addition, a two-way ANOVA was performed, which also indicated a significant effect of both factors. Error bars represent 1 SEM. Details on statistical testing given in the main text.

In the active condition, the sequence of stimuli in each trial was: presentation of the sound texture, display of the question “Have you noticed a change?” on the screen, a 2-s response window, and then a silent period of uniformly randomized duration (1–2 s; Fig. 1*C*). The experiment consisted of one session with 720 stimuli, lasting for ∼90 min. The sequence of trials was randomized independently for each subject. Subjects were instructed to focus on the auditory stimulation and press a button if they detected a change (see Fig. 2). They were asked to respond after a beep to avoid motor response contamination of the EEG signal. No feedback was provided to the listeners regarding their correct/incorrect choice, to prevent fluctuation of their response criterion. In the passive condition, the sequence of events for each trial was shortened due to the absence of the response window; thus, the experiment lasted ∼80 min. Passive subjects were instructed to focus on the ongoing auditory stimulation, but were not told to listen for anything specific, such as a change. During the experimental procedure, all subjects were constantly monitored via video and live EEG recordings by the experimenter to make sure they remained awake. If signs of drowsiness (characteristic prevalence of α-oscillations, not maintaining body posture, eye closing) were detected, a short break was provided to the subject.

To reduce blinking during the presentation of the acoustic stimulus, subjects were instructed to blink only during the silence period occurring after the response window. This blinking period was indicated by a text message displayed on the screen and, additionally, subjects could take a short break at this point by pressing a “pause” button. Overall, most subjects took either one or two breaks during the whole experiment. The number of breaks was higher in the passive groups, but it never exceeded five. The aim for this self-paced experimental scheme was to provide the participants with direct control in relation to their alertness state.

### Experimental setup

#### Presentation of acoustic stimuli

In all experimental conditions, stimuli were prepared online using MATLAB (The Mathworks), then converted to analog signals and presented directly via high-fidelity headphones (Audio-Technica ATH-AD500). The experimental procedure was conducted using Presentation software (NeuroBehavioral Systems).

#### Registration of EEG signals

EEG recordings were performed with a Biosemi 64-active cap electrodes device arranged according to the 10-20 system (Active Two, Biosemi). In addition, four electrodes were located at the external canthi of both eyes, above and below the right eye and another two were placed on mastoids and recorded simultaneously. Electrode placement was standardized using matched Electro-Caps (Electro-Cap International Inc.). EEG signals were sampled at 1024 Hz.

### Data analysis

Data were preprocessed using Brain Vision Analyser 2 (BVA; Brain Products) and then exported to MATLAB for further analysis. First, noisy or missing channels were interpolated using the spline interpolation method (order: 4, degree: 10, λ: 10^−5^). Independent component analysis (Makeig et al., 1996), implemented in BrainVision Analyzer 2, was applied to correct blink artefacts.

**Figure 3.**
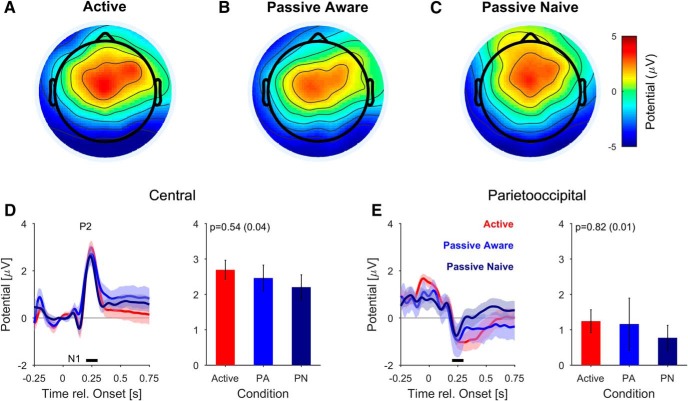
Onset responses are similar across levels of task engagement. ***A–C***, The scalp distribution of the P2 component (200 ms after stimulus onset) is centered with a slight asymmetry toward the front. Since the different stimulus conditions are all matched at the onset, the data were averaged over all stimuli. The scalp topography in the passive–aware conditions mirrors the active potential (***B***). In the passive-naive conditions, the topography was still centered, but slightly broadened toward frontal locations, probably due to uncontrolled blinks (***C***). Overall, the topographies were not statistically different (see text for details). Subject involvement was supported by their relative level of α-band activity (Extended Data Fig. 3-2). ***D***, The central potential, typically associated with auditory-cortex activity (Nie et al., 2014), exhibited a classical response sequence after stimulus onset, with a negative component (N1, 150 ms) followed by a positive component (P2, 242 ms), both slightly delayed compared to classical latencies for pure tones (left, average over electrodes Cz, C1 and C2). The P2 peak size decays slightly but nonsignificantly from active to passive-aware and passive-naive (right, height averaged in [0.2-0.3]s). ***E***, The onset-elicited response in the PO region also displayed a negative deviation around [0.2,0.3]s (left, electrodes Pz, POz), which likely reflects the negative part of the auditory cortex dipole (Extended Data Fig. 3-1) This activity did not differ significantly across conditions. Error bars in all plots indicate ±1 SEM. Effect size is given in parentheses after the *p* value.

All signals were referenced offline to the common average. Next, signals were downsampled by a factor of 8–128 Hz, low-pass filtered (4th order Butterworth, using the MATLAB function butter) at 30 Hz, and high-pass filtered (15th order Chebyshev filter, using the MATLAB function cheby2) at a very conservative 0.1 Hz. Epochs were extracted for each stimulus condition, which spanned from 500 ms before stimulus onset to 1500 ms after stimulus offset. Epochs were rejected if any artifact exceeding ±500 μV occurred at any time during the epoch. The remaining epochs were referenced to the median voltage across all channels. Further, baseline correction was performed by subtracting the median voltage in a 100-ms window before stimulus onset (400–500 ms; see Results for reasoning) or the change in statistics (0–100 ms), respectively, for the different alignments. Scalp-distributions of EEG were computed and visualized using the EEGLAB function topoplot (Fig. 3*A–C*).

As in previous studies of evidence integration (O’Connell et al., 2012; Boubenec et al., 2017), we quantified the slope and height (i.e., potential size w.r.t. baseline) of the potentials in their relation to properties of the stimulus. If evidence is summed by the brain, one expects a linear increase of brain potential with time, with its slope determined by the amount of evidence (Boubenec et al., 2017). The slope was measured for a limited temporal window (400–650 ms after stimulus change) to avoid fusion of the measurement with the flat phase directly after the change, and after reaching the peak (similar to (O’Connell et al., 2012). The potential height was measured as the average within a window centered on the average peak of all conditions after the stimulus onset/change (window location and duration indicated in each panel).

### Source analysis

In addition to scalp level analysis we also performed source analysis to localize the generators of the measured signals. While this analysis has known uncertainties, it still provides important cues to the signal origin (Tarkka et al., 1995; Hegerl and Frodl-Bauch, 1997; Jemel et al., 2002) and presently highlights parietal over frontal contributions to the change response. Single subject trial-averaged data were transferred to BESA (version 5.2, BESA GmbH) for source analysis of the onset and change responses. BESA uses discrete equivalent current dipoles to model intracortical sources of neural responses. The algorithm modifies the position and orientation of the current sources (or pairs of sources) iteratively until a maximal amount of variance as measured by the EEG electrodes is explained (Scherg and Picton, 1991). The model output contains the dipole location and orientation as well as the resulting temporal source wave form.

We applied two separate BESA source models (Simpson et al., 1990) to localize the intracerebral sources of the AEP components elicited by (1) overall onsets (from silence to texture; Extended Data Fig. 3-1) and (2) change responses (transition from one texture to another; Fig. 9).

10.1523/ENEURO.0090-18.2018.f3-1 Extended Data Figure 3-1 The parietal potential at and before stimulus onset has a visual origin (******A******, ******C******). The auditory response can be well explained by two orthogonal dipole sources located in the auditory cortex (******A******, ******B******). The parietal dipole (******D******) remains quiet at the onset of the stimulus, indicating that the negative potential around 240 ms can indeed be accounted for by a mixture with the auditory cortex source(s) (see ******B******). The residual variance of this estimate was 1.48%. Download Figure 3-1, TIF file.

For modeling overall texture onsets, we pooled all mixing coefficient conditions across all subjects to maximize the signal-to-noise ratio of the auditory evoked potentials. A principal component analysis was calculated over the last 50 ms to compensate for drift artefacts. Since a fixation cross appeared before sound onset, a pair of symmetric dipoles was employed to model activity at about -250 ms in the visual cortex (Extended Data Fig. 3-1). Location and orientation of these two dipoles were fixed to separate the specific auditory responses from remaining visual activity. Then, the auditory N1-P2 response (peak-to-peak interval) was fitted with a symmetric pair of dipoles, placed on Heschl’s gyrus, BA41. Those source waveforms exhibited a maximum negative deflection at ∼178 ms in the left and right auditory cortex. These dipoles were kept active, fixed in location and orientation, while a second dipole pair, placed on lateral surface of STG, BA41, was introduced to the model. Lastly, a pair of parietal dipoles was added to the model (location consistent with the second model described in the next paragraph). The same strategy was repeated for each subject and the resulting waveforms were averaged. The residual variance of this grand-average six-dipole model was 1.48%.

For disentangling the responses at the change in statistics, we extended the source model of the sound onset (Fig. 9). Since the change-specific auditory evoked potential was fairly small due to the balanced energy before and after the texture change, the tangential and radial dipoles which accounted for the N1-P2 responses of the auditory onset models described above were used without any further fitting to represent change related activity in auditory cortex. When averaged, this dipole solution resulted in a residual variance of 4.2% (0–2000 ms). The remaining late responses were accounted for by introducing four symmetric dipole pairs: the first pair covered the prominent monopolar deflection from 400–1000 ms generated at parietal sites. A second pair of dipoles was introduced to model the monopolar deflection ranging from 500 to 1300 ms, which was found to be located in the frontal lobe. Finally, the remaining bipolar activity (peak-to-peak at ∼400–900 ms) was captured by a dipole pair which was located lateral to the auditory cortex and one pair in visual cortex. The sequence of inserting the dipoles did not change the attribution of their source activations qualitatively, since the entire model is always refitted with the entire set of dipoles. The introduction of these four additional dipole pairs resulted in a reduction of the residual variance from 4.2–0.64% (0–2000 ms). to allow statistical testing across stimulus parameters, the individual subject data were then analyzed with this set of fixed dipole locations, adapted only slightly for single subject location of auditory cortex and allowing the dipole orientation to differ between subjects. This resulted in different activations of the dipoles per subject. For visual display, all single subject source waveforms were exported to MATLAB and averaged.

### Statistical analysis

Generally, nonparametric tests were used, i.e., Wilcoxon’s signed ranks test for two group comparisons. When data were normally distributed, we checked that statistical conclusions were the same for the corresponding test, e.g., *t* test. One-way analysis of variance was computed with the Kruskal–Wallis (nonparametric) test or ANOVA (parametric, in the case of normally distributed data), respectively; two-way analysis of variance was computed with the two-way ANOVA test (due to the lack of suitable two-way nonparametric tests). Effect sizes were computed as the variance accounted by a factor, divided by the total variance, and are always provided in parentheses after the *p* value in each panel. Error bars indicate 1 or 2 SEM depending on the figure. *Post hoc* pairwise multiple comparisons were assessed using Bonferroni correction. All statistical analyses were performed using the statistics toolbox in MATLAB (The Mathworks).

## Results

We investigated the neural representation underlying the detection of change in the statistics of natural acoustic environments and its dependence on the level of task engagement. A group of 18 subjects was asked to detect changes in statistics, while a partially different group of 18 subjects listened passively to the same set of stimuli. Nearly half of the passive subjects were chosen to overlap with the active group; therefore, two passive conditions (passive-aware and passive-naive) will be analyzed separately. The statistics of the stimuli changed by a randomized amount at a randomized time. We evaluated the size and slope of the most salient evoked potentials (as in O’Connell et al., 2012; Boubenec et al., 2017), with respect to the level of task engagement; we also estimated the underlying cortical sources.

### Integration time and change size improve reaction time and performance

Natural acoustic textures are characterized by their statistics, and longer exposure improves their recognition (McDermott et al., 2013). We first verified this relationship for changes in statistics occurring unexpectedly during continuous presentation. Consistent with this hypothesis, we found that an increased exposure to the first texture was correlated with shorter reaction times and improved performance (Fig. 2). Performance is here quantified as the rate of button presses, which are correct for signal trials (detection rate with mixing coefficient > 0) and incorrect for catch trials (correct rejection rate with mixing coefficient = 0).

Reaction times decreased significantly with later change times and larger change sizes [Fig. 2*A*; *p* < 0.001 and *p* < 10^−10^, two-way ANOVA with factors change time (df = 2) and change size (df = 3), *n* = 18, the interaction between the factors was nonsignificant (*p* = 0.012)]. Note, that responses to the no-change condition (Mixing Coefficient = 0, light blue) constitute only incorrect responses, but it demonstrates that in the presence of no evidence, the false alarm decision is delayed as well. Within a change size level, reaction times exhibit a nonsignificant decrease [Kruskal–Wallis, *p* values see Fig. 2 legend (df = 2), *n* = 18]. Conversely, reaction times decreased significantly with change size within each change time [*p* < 0.001 for each, Kruskal–Wallis (df = 3), *n* = 18]. Reaction times were normalized to the median of each subject to prevent general reaction time differences and to overrule differences due to stimulus parameters.

Detection rates significantly improved with later change times and larger change sizes (Fig. 2*B*; *p* < 10^−10^, two-way ANOVA as above, *n* = 18). Within a change size level, dependence on change time was highly significant [Kruskal–Wallis, *p* values see Fig. 2 legend (df = 2), *n* = 18] except for the catch trials. The lack of significance here indicates that subjects did not expect longer trials to be more likely to contain a change. Detection rates also increased with change sizes within each change time [*p* < 10^−10^ for each, Kruskal–Wallis (df = 3), *n* = 18]. The interaction between the factors was significant as well (*p* = 0.018).

Overall, change time and change size had the expected effect of modulating the subjects’ ability and speed to detect changes in stimulus statistics.

### Task-irrelevant onset of auditory textures is not reflected in the parieto-occipital (PO) potential

Onsets and offsets of auditory stimuli typically produce a distinct response in the auditory cortex which is discernible in EEG recordings (Anderer et al., 1996). We first performed their signal analysis on the scalp level, before stepping toward source analysis (Extended Data Fig. 3-1; Fig. 9). The evoked potential is temporally composed of a negative deflection at ∼100 ms (‘N1’) and a larger positive potential at 200–250 ms (‘P2’). These classical responses are most clearly reflected with these polarities in the central electrodes (Nie et al., 2014), due to bilateral summation (Fig. 3*D*, averaged over all stimulus conditions). Given the scalp topography, we used a central set of electrodes (C1, Cz, C2, corresponding to the procedure applied by Nie et al., 2014).

10.1523/ENEURO.0090-18.2018.f3-2 Extended Data Figure 3-2 Distribution of spectral power in different levels of task involvement. Active subjects show barely elevated power in the α-band (red, *n* = 18). The passive-aware subjects showed elevated α-band activity around 10–15 Hz (maroon, *n* = 8). The passive-naive subjects showed elevated α-band activity around 9–14 Hz (black, *n* = 10). Spectra were computed from channel Oz. The difference between the three conditions was significant (*p* < 0.001, two-way ANOVA, on frequency (df = 5, range 9–15 Hz, vertical lines) and condition, df = 2). The elevated α-band can be taken as an indication for a reduced level of task engagement in both passive groups compared to the active group. The power spectra were computed in the 1.5 s preceding the start of the stimulus to avoid contributions of the task-related ERPs. Outside the depicted frequency range, the spectra were quite similar. Error hulls represent ±1 SEM. Download Figure 3-2, TIF file.

The average potential revealed a classical N1/P2 complex (extrema at 155 and 240 ms, respectively), which did not differ across conditions as expected (Fig. 3*D*). While the passive-aware topography (Fig. 3*B*) mirrored the active (Fig. 3*A*) one, the passive-naive (Fig. 3*C*) topography was broadened toward frontal areas, possibly due to blink residuals. The shape of the central potentials did not differ significantly across the three conditions [*p* > 0.12 for the conditions, two-way ANOVA on channels (df = 63) and conditions (df = 2), normalized by their norm across channels, *N* = 17]. More local comparisons did also not suggest a significant difference in shape [e.g., AFz compared between passive-naive and the other two conditions, Kruskal–Wallis on conditions (df = 2) and *post hoc* test, Bonferroni corrected, *p* > 0.05]. The P2 peak did also not differ significantly across task involvement (Fig. 3*D*; *p* = 0.54, one-way ANOVA, *N* = 17).

**Figure 4.**
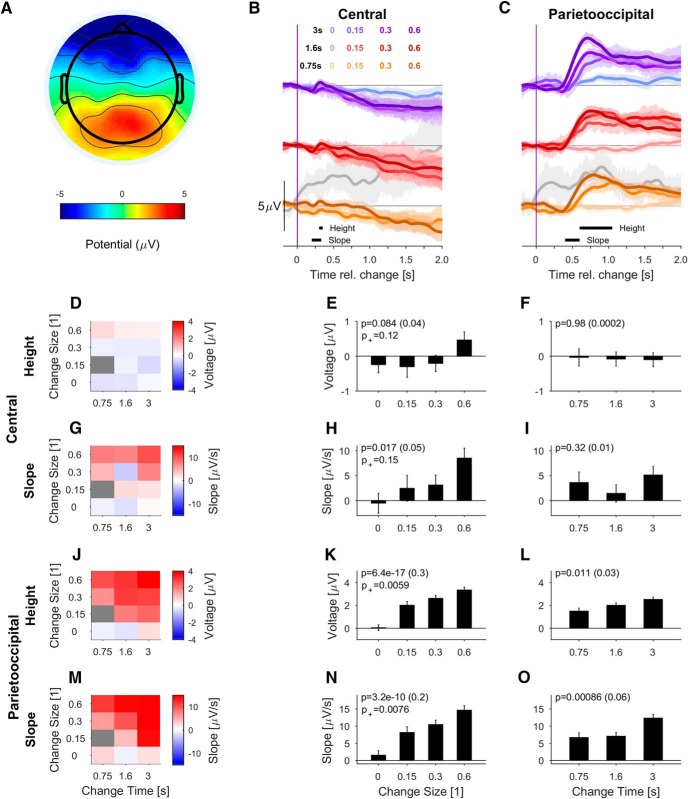
PO potential depends on stimulus evidence and integration time during task engagement. ***A***, Scalp topography of change-elicited activity at 0.7 s post-change exhibits a single peaked positivity over the PO regions for correct detection (hit) trials. Only trials with the lowest difficulty, i.e., MC = 0.6 were included for clarity of display. ***B***, In the central electrodes (auditory cortex associated, as in Fig. 3), the change in statistics leads to a delayed ERP only for the longest change times and the largest mixing coefficients. Data are aligned to change time and averaged over hit trials only. Black lines at the bottom indicate window selected for the analysis of height (300–350 ms) and slope (200–330 ms). The responses for different change times are shifted vertically to avoid crowding. Gray trials indicate conditions with <10% of response percentage and correspondingly more variability. If all trials are included, variability is comparable between conditions (Extended Data Fig. 4-1). ***C***, The PO potential (electrodes as in Fig. 3) first turns slightly negative and then becomes strongly positive, lasting until the end of the sampling period of the second texture. The potential's height (600- to 1000-ms window) and slope (400- to 600-ms window) depended significantly on change time and size. Colors as in ***B***. The subsequent panels show the dependence of the slope and height of the respective potentials for all stimulus parameters (left column, color bar represents slope or height) or in relation to change size (middle) and change time (right) only. ***D****–****F***, Height of the central potential did not depend significantly on change size or change time (see ***B*** for measurement range). ***G-I***, The slope of the central potential depended significantly on change size, while it did not depend on change time (see ***B*** for measurement range). ***J***, The PO potential's height also depends systematically on change time and size (see ***C*** for measurement range). ***K***, The height of PO potential varied significantly with change size both if catch trials are included or not. ***L***, The PO potential's height dependence on change time was also significant. ***M***, The slope of the PO potential increased with both change time and change size. ***N***, Change-related build-up in PO potential is significantly faster with increasing change size. ***O***, The PO slope dependence on change time was highly significant. All error bars indicate ±1 SEM, and *p* values of the two-way ANOVA for the factor on the *x*-axis are denoted in the figure, all tests are described in the main text. p^+^ is the significance when leaving out the no-change condition. Effect size is given in parentheses after the *p* value.

Because of its relation with evidence integration, we also analyzed a central-posterior set of electrodes (related to O’Connell et al., 2012; Kelly and O’Connell, 2013; Boubenec et al., 2017; see next section). Based on the topography shown in Figure 4*A*, we chose a set of parietal-posterior electrodes (Pz, POz), which are located a bit more posterior compared to the aforementioned studies (see also Discussion). We thus term this potential PO, although it appears to be very related to the CPP (O’Connell et al., 2012; Kelly and O’Connell, 2013; Boubenec et al., 2017).

10.1523/ENEURO.0090-18.2018.f4-1 Extended Data Figure 4-1 Same analysis as in Figure 4 but for all trials. Nomenclature identical to Figure 4. Demonstrates that the variability was very similar across conditions; however, the number of trials per condition differed across hits and misses. Note that the strong dependence of slope and height on change size and change height is due to the larger number of miss trials for small change sizes and change times. Download Figure 4-1, TIF file.

Before and around stimulus onset, the PO potential is dominated by the visual potential evoked by the appearance of a fixation cross 500 ms before sound onset (Fig. 3*E*; for a source separation of the different contributions, see Extended Data Fig. 3-1). Therefore, the PO potentials were baselined at 500–400 ms before the onset of the acoustic stimulus to avoid confounding the reference with the visual potential. The sound onset also appears to be visible in the PO electrodes at the same latency (250 ms) as in the central electrodes. Similarly, its size does not differ across task involvement (*p* = 0.82, one-way ANOVA). We hypothesized that this potential reflects the activation of auditory cortex, rather than a parietal source, which was confirmed using analysis of neural sources (Extended Data Fig. 3-1*B–D*). Subsequently, the PO potential returns to baseline.

In summary, the neural response to the onset of a natural acoustic texture shares properties with the onset response to other sounds, although the N1/P2 components are delayed by ∼40 ms with respect to pure tones. The sound onset reflected in the PO potential appears to stem from the auditory cortex source.

### Task-relevant transition between auditory textures elicits a graded PO potential

Next, we analyzed the response at the same scalp locations to the change in statistics for natural auditory textures (Fig. 4). In correct detection trials, the central electrodes exhibited no or much smaller responses than to stimulus onset. On the other hand, the PO electrodes showed strong responses for all change sizes and change times.

The central potential could be visually separated into two phases. In the early phase, large change sizes evoked an ERP whose shape was reminiscent of the classical N1/P2 complex, but showed longer latencies than those observed at stimulus onset (∼320 ms for P2; Fig. 4*B*). Note that the physical change in the stimulus only explains 20 ms via the speed of transition between the textures (for 10–90%), see Eq. 2). In the late phases, a slow negative potential built up, which stayed negative for >1 s (for more details, see Fig. 8). This finding is in line with the N1-P2 acoustic change complex identified in response to change in periodicity (Martin and Boothroyd, 1999). The height of the early component, measured as the potential w.r.t. baseline at the average peak time (Fig. 4*E*,*F*; *p* = 0.084 and *p* = 0.980, respectively) did not reach statistical significance even for large changes in spectral properties (mixing coefficient = 0.6). Its slope depended significantly on change size (Fig. 4*H*; *p* = 0.017), but not change time (Fig. 4*I*; *p* = 0.32).

Conversely, the PO potential exhibits a large, late positive response (peak at or after 700 ms post-change; Fig. 4*C*) for all mixing coefficients >0. The scalp topography of this potential broadly spans the PO region [Fig. 4*A*; for clarity, only the largest change size (MC = 0.6) conditions are shown here], and is consistent in shape with the P3 response (Dreo et al., 2017). The slope and height of this peak depends strongly on change size and change time [Fig. 4*J–O*; two-way ANOVA with factors change time (df = 2) and change size (df = 3), K: *p* = 6.4e-17, L: *p* = 0.011, N: *p* = 3.2e-10, O: *p* = 0.00086, *N* = 17]. If the no-change condition was removed, the dependence persisted for both height and slope (indicated as p^+^ in the same panels). In Figure 4*O*, only the slope for change time = 3 s differs significantly from the smaller change times (*post hoc t* test, *p* = 0.004 and *p* = 0.003, respectively, for the comparison with change time = 0.75 and 1.6 s). No significant interactions were found between change time and size.

**Figure 5.**
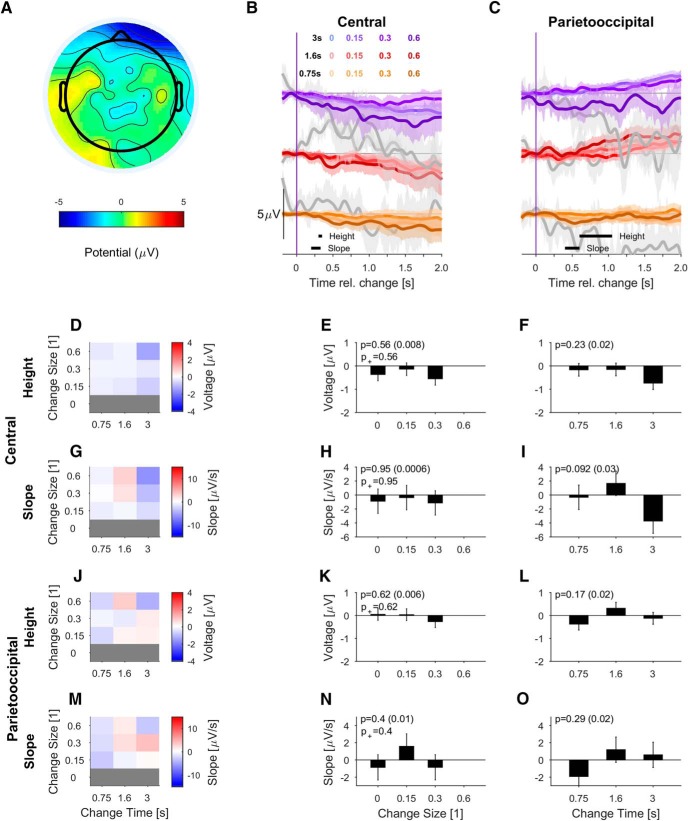
Same analysis as in Figure 4 but for misses. Nomenclature identical to Figure 4. No dependencies were found, except for a central potential, slight, but inverted dependency for height (with a borderline *p* value). Gray curves indicate conditions containing <10% of possible trials.

In contrast, the PO potential was nonexistent for the no-change trials (Fig. 4*K*,*N*). In trials in which the change was not detected (misses), the late positive PO potential was also completely absent (Fig. 5, see Discussion for an interpretation of these results). Note, that the different variability between conditions stems from the number of trials entering them based on human performance. If all trials of a condition are considered, instantaneous variability across the conditions is much more uniform (Extended Data Fig. 4-1). The PO potential also exhibited an initial negative potential, peaking at ∼350–400 ms post-change, whose size at first glance appears to vary with change time and size, but neither were significant [*p* > 0.05, two-way ANOVA w.r.t. change time (df = 2) and size (df = 3) for the window 300-400 ms post-change, *N* = 17]. No significant interactions were found between change time and size.

Interestingly, change-evoked activity in parietal regions is reflected negatively in frontal channels (Fig. 4*A*). Based on the dominant contribution of the parietal sources (Fig. 9*F*), we focus here on the analysis of the parietal electrodes but acknowledge that the parietal electrodes may also partially reflect a frontal source activation.

Finally, since subjects responded after the sound ended, direct motor activation was excluded in the present analysis. However, the influence of the response-time distributions is relevant (for details, see Discussion).

In summary, the central and PO potential exhibited opposite dependencies on stimulus onset and change in statistics of the natural texture. While the central potential is evoked by basic transitions in level or spectrum, e.g., at the onset or during large change sizes, the PO potential's size and slope are mostly not affected by stimulus onset but depended strongly on both evidence amount and accumulation time. The observed PO activity is therefore likely related to the CPP reported by O’Connell et al. (2012; for more details, see Discussion). Next, we asked how task-involvement influenced the dependence of these potentials on the stimulus.

### PO dependence on change properties depends on task knowledge

In the passive paradigm, subjects listened to the stimuli without responding to any perceived changes. This condition mimics the everyday situation of listening to an ambient soundscape without a strong expectation of a change or attention on its detection. The passive group (*n* = 18) was subdivided into subjects that had performed the active task before (passive-aware, *n* = 8) and naive subjects (passive-naive, *n* = 10). Although they did not have to behaviorally respond to the embedded changes, the former group was acquainted with the stimulus and task context. We expected that subjects would get bored with the listening “task” and barely pay attention to the stimulus. Consistent with this view, the three groups displayed significantly different power spectra (Extended Data Fig. 3-2; computed for electrode Oz), with active subjects displaying barely any α-band activation (red), whereas both the passive-aware (brown) and the passive-naive (black) conditions showed elevated α-band activity. We thus investigated to what extent the PO potential was specific to task engagement.

**Figure 6.**
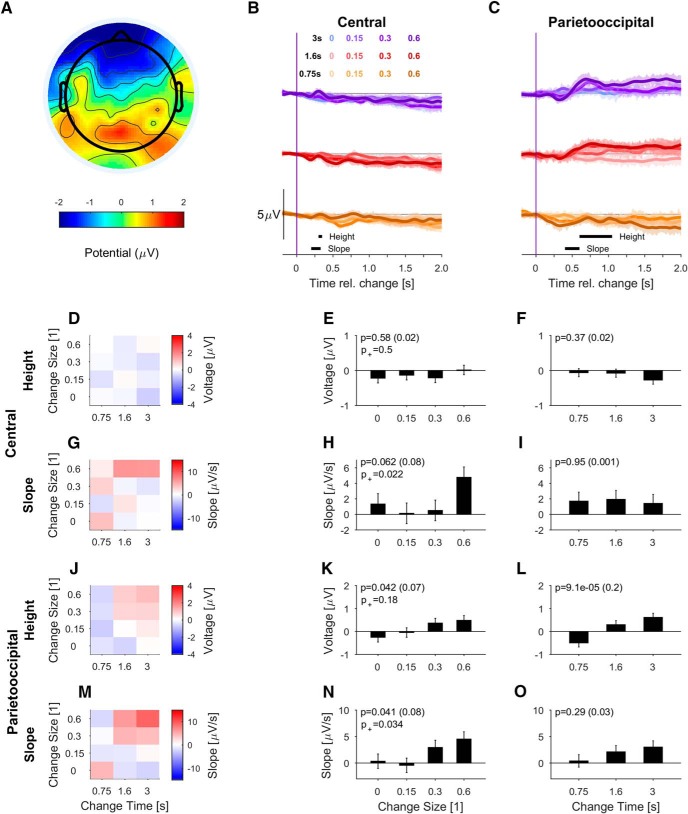
The PO potential is reduced but stays stimulus dependent for passive-aware subjects. ***A***, The post-change scalp topography (600–700 ms) is dominated by posterior components, but the overall potential size is much smaller (only trials for MC = 0.6 included for display); however, note the scale difference in comparison to Figure 4. ***B***, In the central electrodes, the change in statistics elicited a small ERP complex (positive peak ∼320 ms) for the longest change times and largest change sizes (colors as in Fig. 3, see legend). ***C***, In the PO electrodes, the change in statistics also led to a slow negative-positive potential, reaching a plateau around 700 ms. ***D****–****F***, The height of the central potential neither depended on change size nor time. ***G–I***, The slope of the central potential showed a borderline significant dependence on change size, but not change time (measurement range: 200–330 ms). ***J***, The PO potential's height varied systematically with change time and size. ***K***, The PO potential was significantly larger for bigger change sizes; however, the size of the potential was much smaller than in the active condition, corresponding to the change in topography. ***L***, The PO potential also increased slightly but significantly in height with change time (see Results for interpretation). ***M***, The slope of the PO potential exhibited a similar dependence on change size and time as in the active condition (compare to Fig. 4*G*). ***N***, The PO slope is significantly steeper as a for larger change sizes. ***O***, The PO slope displayed a nonsignificant tendency to be faster for longer change times. Conventions as in Figure 4.

**Figure 7.**
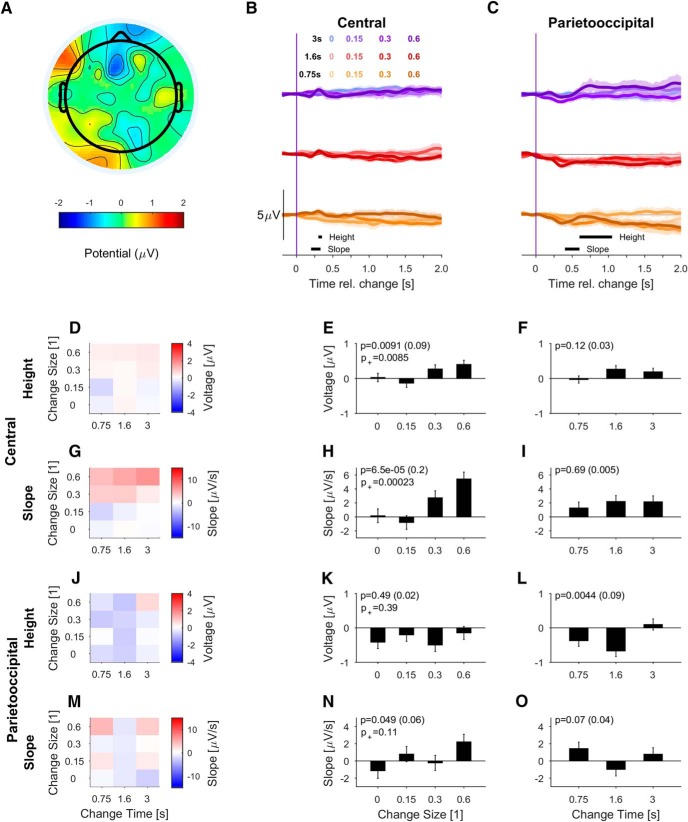
In the passive-naive group the PO potential exhibits much weaker but partially significant dependence on change size and time. ***A***, The post-change scalp topography (600–700 ms) exhibits a more diverse shape than in the active or passive-aware conditions (only trials for mixing coefficient equal 0.6 included for display). The scale bar was kept the same as in Figure 5, to emphasize the difference in potential size. ***B***, In the central electrodes, the change in statistics again elicited a small ERP complex (positive peak around 320 ms), as in the other conditions (colors as in Fig. 3, see legend). ***C***, In the PO electrodes, the change in statistics lead to residual potentials with much more diversity between stimulus properties than in the other conditions. ***D****–****F***, The height of the central potential depended significantly on change size but not on change time. Measurement range (300–350 ms post-change) is shown in ***B***. ***G–I***, The slope of the central potential also depended significantly on change size but not on change time. ***J–L***, The PO potential's height depends significantly on change time, however, with a nonmonotonic progression. ***M–O***, The PO potential's slope depends significantly on change size; however, this effect is driven by the last condition with MC = 0.6. Conventions as in Figure 4.

In the passive–aware group, the post-change scalp topography was dominated by a PO positivity (at 700 ms; Fig. 6*A*) as in the active case. However, it was smaller and broader than the active group. In the passive-naive group, the topography was much less localized (Fig. 7*A*). The passive-aware properties of the PO potential depended in a similar manner on the parameters of the stimulus (Fig. 6*J*,*M*) as in the active case (compare with Fig. 4), i.e., height and slope increased with change size (Fig. 6*K*,*N*), and height increased with change time (Fig. 6*L*). The dependence of height on change time is, at first glance, surprising; however, it likely stems from a combination of (passively perceived) “hit” and “miss” trials, which leads to a dependence on change time due to the differential contributions of these outcomes at different change times (compare Extended Data Fig. 4-1). In contrast, the early (240 ms) central potential showed no dependence (Fig. 6*D–I*).

The passive-naive group showed a more diverse picture of dependence with multiple nonmonotonic dependencies. The clearest dependence (Fig. 7*G*) was that of the slope of the early potential (P2-like) on change size (Fig. 7*H*; *p* values). Further, the PO potential exhibited borderline significance for slope in relation to change size, mostly driven by the largest change size (Fig. 7*N*).

In summary, a behavioral response to the change was not necessary for observing a stimulus dependent, post-change PO potential. In the passive-aware subjects, the potentials were reduced but maintained a similar relationship to the stimulus properties as in the active condition. Even in the passive-naive group, some dependencies remained, however, with much smaller sizes.

### Change-evoked parietals are scaled by task-engagement and task-knowledge

**Figure 8.**
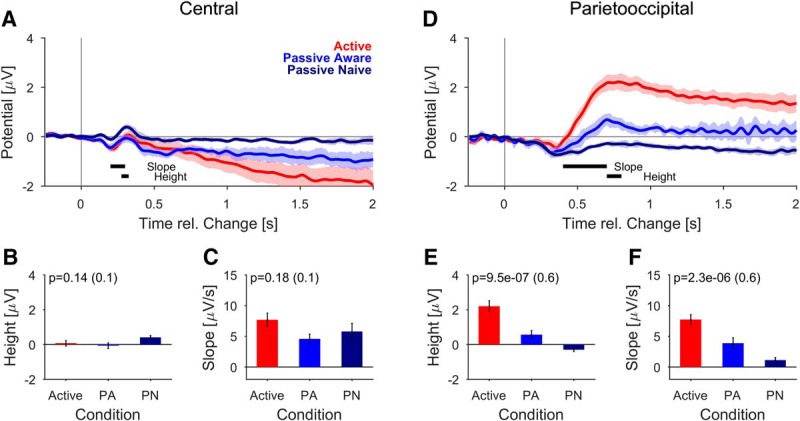
Task involvement scales change-related potentials ***A–C***, Change-time aligned, central potentials exhibited different profiles across task involvement. The initial P2-like response (peak at 313 ms) did not differ significantly across involvement levels [height (***B***), slope (***C***)]. However, the potentials diverged strongly thereafter, with increasingly negative slopes for more active involvement. Data averaged over all change times and the two largest mixing coefficients (0.3,0.6). ***D–F***, The PO potentials exhibited clear differences in slope (***E***) and height (***F***) across different levels of task involvement. For the passive-naive condition, the potential appears to be a combination between a lasting negative potential in combination with a more transient positive potential added. All error bars indicate ±1 SEM, and *p* values are denoted in the figure, all tests are described in the main text. Effect size is given in parentheses after the *p* value. Same electrodes as in Figure 3.

The analysis above focused on the dependence of the neural response on stimulus properties but did not compare them side-by-side for different levels of task involvement. This comparison is important to analyze the degree to which task involvement modulates cortical representation of the accumulation of sensory evidence. For this purpose, we compared the central and PO potentials, averaged over the two largest change sizes (MC = 0.3 and 0.6; Fig. 8).

Comparing the response patterns across levels of task involvement, the central potential appears to be composed of two overlapping constituents, which was not as clearly discernible in Figure 4. First, the P2-like, positive potential occurs (Fig. 8*A*; peak: 313 ms), overlapping with a longer lasting, nearly linear decline in potential. While the P2-like potential does not differ significantly in shape across task involvement levels (Fig. 8*B*: height, *p* = 0.14; Fig. 8*C*: slope, *p* = 0.22, one-way ANOVA), their long-term behavior differs significantly (*p* = 0.038, one-way ANOVA across task involvement level, measured in the time window of 1.5–2.5 s).

**Figure 9.**
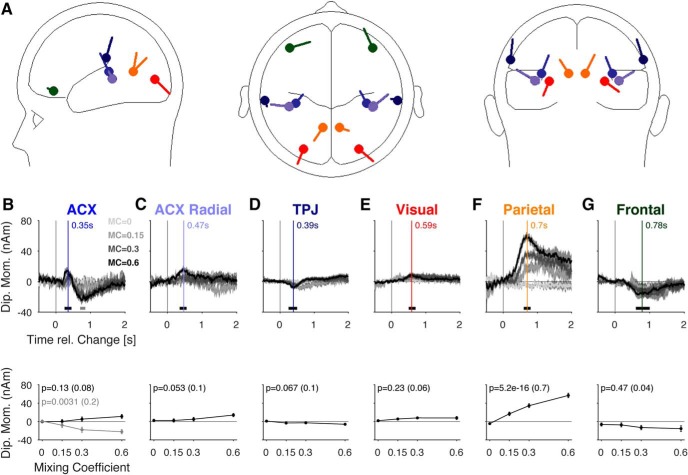
Contribution of the different sources to the change related response. ***A***, The scalp potential was well approximated [residual variance (0–2000 ms) = 0.64%] by a mixture of six sources in each hemisphere, activating at different times after the change. The sources are located in the auditory cortex (blue, light blue, ***B***, ***C***), the temporo-parietal junction (dark blue, ***D***), the visual cortex (red, ***E***; provided for completeness), the medial-parietal cortex (orange, ***F***), and finally the frontal cortex (green, ***G***). All sources were estimated as bilateral pairs; however, potentials in ***B****–****G*** show averages across pairs. ***B***, The first source activation was located close to the auditory cortex (blue in a ***A***). It exhibited a significant dependence on change size only around 750 ms (bottom, gray, analysis window: 700–850 ms), while the P2-like potential (peak: 350 ms, vertical blue line, correspondingly in the following panels) was borderline insignificant (bottom, black, window: 250–450 ms). ***C***, As before, we estimate a radial component of the AEP, which exhibited a borderline significant dependence on mixing coefficient (bottom, window: 350–550 ms). ***D***, The activation of the temporo-parietal junction was very small and did not contribute significantly (window: 250–500 ms). ***E***, The contribution from the visual source was nonsignificant (window: 500–700 ms). ***F***, The activation in the parietal cortex exhibited a clear and significant dependence on the mixing coefficient. While the onset time remained similar across change sizes, the peak time was slightly delayed for lower change sizes, similar to potentials the scalp level (window: 600–800 ms). ***G***, A further pair of sources was localized in the frontal cortex, which, however, did not show a significant dependence in their size on change size (window: 600–1000 ms). Error hulls and bars indicate ±1 SEM over *n* = 17 subjects, and only 3-s change time is shown. Effect size is given in parentheses after the *p* value.

The PO potential exhibits a very strong dependence in height (Fig. 8*E*; *p* < 0.001, one-way ANOVA) and slope (Fig. 8*F*; *p* < 0.001, one-way ANOVA) on task involvement. Across these levels there is a common initial negative potential (Fig. 8*D*), putatively reflecting the auditory cortex contribution (compare also Fig. 9), followed by a superimposed positive potential.

In summary, we find the rapid, P2-like component in the central electrodes to not differ across task involvement levels. in contrast with the salient dependence in the later phase at both central and parietal electrodes. In particular the difference in slope in the late central potentials requires an analysis of the underlying sources to decide whether this is supported by auditory cortex activity.

### Change-evoked potentials are composed of auditory, parietal, and frontal sources

Scalp level potentials provide a direct and unbiased readout of cortical activity; however, they typically do not allow immediate insight into the underlying neural sources. We therefore performed source localization of the post-change potentials to estimate the source locations that generated the observed scalp potentials (Fig. 9). Despite the possible, well-known pitfalls of source analysis, we consider the results valuable for identifying target regions for neuron-level neurophysiological investigation. Source activations were estimated based on single subject trial-averaged data (for details, see Materials and Methods).

The first activation in response to the change was localized in the more medial regions of auditory cortex, although latencies exceeded those observed for simple N1/P2 complexes at stimulus onset (compare Figs. 9*B*, 3; 240 vs >320 ms), similar to latency changes observed for tasks of different complexity before (Chait et al., 2008). This P2-like peak did not scale strongly with mixing coefficient (different shades of gray, change time = 3 s, one-way ANOVA across mixing coefficients, *N* = 17, *p* = 0.057). Another negative dipole moment was observed at much larger latencies (>∼750 ms), not observed at the central electrodes, which scaled significantly with mixing coefficient (p = 0.0031, as above). The radial component's first peak occurred later (∼500 ms; Fig. 9*C*), and exhibited borderline dependence on change size (p = 0.023). Temporoparietal junction (TPJ) activation exhibited a similarly low level of dependence (Fig. 9*D*; *p* = 0.067). Visual cortex displayed no significantly changed size in potential (Fig. 9*E*; *p* = 0.68). The neighboring internal parietal cortex exhibited the most distinctive activation (Fig. 9*F*; *p* = 2.5 × 10^−15^). Finally, a frontal source was detected, which exhibited an elongated, negative activation with no dependence in response size on change size (Fig. 9*G*; *p* = 0.56).

Hence, the estimated source activations suggest an activation of multiple sources in response to the change. The most dominant and stimulus-dependent activations were found in the medial parietal cortex (precuneus) and a late response in auditory cortex. The former was generally predicted by the scalp potentials but appears to be located more internal than expected (see Discussion about area LIP). The latter was not identifiable on the scalp level but suggests a relevant contribution of the (higher) auditory cortex to evidence integration in auditory decision making.

## Discussion

We investigated the neural representation of dynamic change detection in naturalistic textures, a task which requires the accumulation of sensory evidence for a change in statistics. We identified a multistage representation of change-related evidence involving the auditory cortex, precuneus (medial superior parietal cortex) and frontal cortex. Only the late potentials in the precuneus and auditory cortex correlated with the accumulated amount of sensory evidence. Across different levels of task-engagement, the size of the late auditory and parietal potential scaled markedly, however, retaining a reduced scaling with change-related sensory evidence in the passive conditions.

### Change detection and accumulation of sensory evidence

A violation of statistical regularity on the level of spectro-temporal statistics cannot rely on the detection of a single, transient event. These occur frequently in natural textures, but do not indicate relevant changes. An actual irregularity can only be detected by integrating widely across the spectrogram, i.e., integrating distributed evidence. In the present study we investigated the neurophysiological response to changes in complex statistics. A late, parietal component of the ERP was identified, whose slope and size correlate with the accumulated amount of sensory evidence about the change in statistics, which was subsequently localized to the precuneus.

In a related study using different, artificial stimuli, we provided direct support for this interpretation of the PO potential using an immediate response paradigm: the PO potential (termed CPP in that study) exhibited typical signs of evidence integration, such as slow accumulation-to-threshold dynamics (∼1-s time scale) and scaling in slope with the amount of evidence (Boubenec et al., 2017), consistent with previous studies (O’Connell et al., 2012; Kelly and O’Connell, 2013). The present study generalizes this paradigm to naturalistic textures and assess task-involvement.

While the present design precludes an alignment on response times, it excludes the contributions from motor potentials and therefore allows localization of the generating sources. One caveat is, however, that the temporal distribution of detection times will depend on change saliency, which will lead to a widening of change-locked averages i.e., especially for the low change size conditions. Such a dependence of PO height on change size has also been found in immediate-response studies (O’Connell et al., 2012; Boubenec et al., 2017), which may result from continued integration between decision commitment and decision execution. Consistently, the PO potential was absent in trials with unsuccessful evidence integration (misses; Fig. 5).

We found the PO potential to increase with change time. This appears to be in conflict with our previous results (O’Connell et al., 2012; Boubenec et al., 2017), which indicated a reduction as a function of time. However, this difference can be explained by different speed-accuracy trade-offs between the two task designs, i.e., immediate response in a short time window versus delayed report without time pressure. When the response is delayed, there is no urgency to respond and hence evidence integration can continue and reach a higher level of certainty. This allows subjects to take advantage of the long presentation of the post-change stimulus, which was not possible in our previous study. The slope also increased with change time, suggesting that an improved estimate of the baseline statistics accelerates the translation of accumulated sensory evidence to an estimate of a change. While this result aligns with one's intuition, it is interesting to see it reflected in neural activation.

### Neural origin of change-related activity

Previous EEG studies of evidence integration have mostly investigated the neural responses on the scalp level, but have not localized the underlying sources (Garrido et al., 2009; O’Connell et al., 2012; Kelly and O’Connell, 2013). Here, we estimated a detailed source model to account for multiple auditory, visual, parietal and frontal sources, which provides a set of very distinct activation profiles. The present analysis suggests change detection to be based on an activation of auditory cortex, medial parietal cortex (precuneus) and frontal cortex, where the latter does not scale with change size (Fig. 9*C*,*E*,*F*). These results agree with recent findings of cortical sources in target detection tasks (Mulert et al., 2004; Kam et al., 2016) and with the detection of spectro-temporal properties of auditory textures, localized in auditory and association cortices (Overath et al., 2010). Overath et al. (2010) also reported activation in the TPJ, which did not activate robustly here. Moreover, the present dipole locations are in line with an intracranial study by Halgren et al. (1998), who pointed out superior temporal sulcus, lateral orbitofrontal cortex and intraparietal sulcus for P3b generators, showing overlap with the estimated source of the PO potential. This coincides with the role of parietal regions in the accumulation of sensory evidence, in particular visual evidence integration (LIP; Shadlen and Newsome, 2001; Huk and Shadlen, 2005).

Some MEG studies have also performed source localization and suggest frontal sources to be more strongly involved in discovering regularities in statistics than observed presently (Barascud et al., 2016). While we do find long-lasting negative potentials in frontal scalp locations (Fig. 4*A*) as well as a corresponding frontal source activation, the contribution from the precuneus is more substantial and scales well with the hypothesized accumulation process. The long-lasting negative frontal component could be the manifestation of the top-down modulating role of the PFC (Huettel and McCarthy, 2004). The fronto-parietal network has previously been highlighted in the context of evidence integration (Lavigne et al., 2015), while other studies have pointed out a rather exclusive role of parietal regions (FitzGerald et al., 2015).

### Neural responses across different levels of task engagement

The comparison between neural responses in the active and passive paradigms shows that the level of task-engagement strongly modulates the change-related PO potential (Fig. 5). Similarly, previous studies on P3 responses found a correlation between P3 size active reporting, typically by button press (Sergent et al., 2005; Del Cul et al., 2007; Faugeras et al., 2012). A potential caveat of active paradigms is that preparatory motor activity can influence the measured change potential (Doucet and Stelmack, 1999). We, however, consider this not a likely reason for the scaling, as in the present paradigm the peak potential occurred >1 s before the motor response. Previous studies have also deemed this unlikely (O’Connell et al., 2012; Boubenec et al., 2017). Instead, we propose that evidence integration occurs both in active and passive paradigms, as long as the relevance of a change in a given stimulus is known (as it is in the active and passive-aware conditions), while little of this integration remains if the stimuli are meaningless for the subjects (passive-naive), even if the changes are easy to detect in some conditions. While the present study investigated only a single modality, we consider it likely that the task-dependence of the neural response generalizes also to other modalities, scaling the activity in parietal source also for visual tasks, in accordance with the findings of O’Connell et al. (2012).

### Relation to previous work on change detection in complex acoustic stimuli

While previous studies focused on changes in simpler statistics, the present experiment approximates certain real-world challenges that require detecting changes in a complex acoustic stimulus.

In a series of MEG experiments on changes in second-order statistics (Chait et al., 2008), the latency of the N1-like deflection was found to be longer for random-to-regular transitions than for other types of changes tested. We observe a delayed N1/P2 complex, consistent with increased latencies of primary auditory responses for more complex statistical changes (Krumbholz et al., 2003).

Many studies have investigated violations in the structure of acoustic sequences, leading to the well-known mismatch negativity potential (Garrido et al., 2008). This has been observed in a wide range of experimental paradigms, e.g., a sequence of descending tones with an ascending deviant (Tervaniemi et al., 1994), a tone repetition in a random sequence (Horváth et al., 2001), combined rules, such as higher frequency paired with larger level (Paavilainen et al., 2001; Paavilainen, 2013), or even irregularities in rhythms (Vuust et al., 2005) and musical sequences (van Zuijen et al., 2004). The current paradigm is generally similar to MMN paradigms because a violation of regularity occurs. Neural localization of auditory MMN is under debate, with EEG pointing to fronto-medial locations (Näätänen et al., 2012), fMRI to auditory cortical (Opitz et al., 2002; Molholm et al., 2005), and MEG studies to supratemporal sources (Näätänen et al., 2007). However, the present results indicate processing beyond a classical MMN response: (1) long latencies of the change-related ERP (200–300 ms) when compared to classical MMN response (100–250 ms), (2) prominent PO component with long peak latency (∼700 ms), and (3) “automatic” nature of MMN compared with the dependence on change detection of PO. In line with previous studies on sensory evidence integration (O’Connell et al., 2012; Kelly and O’Connell, 2013), we propose that the PO potential reflects this spectro-temporal integration, rather than a transiently discovered violation in regularity.

The present results may be related to the family of P3 response components, typically observed for infrequent, unpredictable changes in the context of decision making (Hillyard et al., 1971; Kutas et al., 1977; for review, see Polich, 2012). As suggested by Twomey et al. (2015), the late and strong PO activity elicited in reaction to changes in statistics could be considered as a general variant of the P3b. For rare targets, P3b was suggested to reflect surprise (Schendan et al., 1997) and the level of uncertainty (Ruchkin et al., 1990), which may both play a role in our paradigm, i.e., change time and change size, respectively. However, the present change-related ERPs have surprisingly long latencies, namely >700 ms instead of the classically observed ∼300–350 ms for the P3b (Polich, 2007).

### Conclusions and outlook

Consistent with our initial hypothesis our results suggest that accumulation of sensory evidence underlies change detection in complex statistical stimuli, evidenced by the behavioral and neural dependence on accumulation (change) time and size. The clearest neural signal for an accumulation process arises from the parietal cortex, probably the precuneus, and also scales in slope and size with properties of the hypothesized accumulation process. Finally, these neural responses were shown to scale strongly with the level of task involvement.

While the current results provide a step toward understanding the processing of natural acoustic stimuli, further research is required to understand which type of statistical changes are most prevalent/relevant and which are tracked passively and thus may induce bottom-up attention. A systematic study of the spectro-temporal statistics of acoustic scenes will be required to provide an interesting stimulus set for additional experiments.

Generally, the present results can be interpreted in the “predictive coding” framework (Barlow, 1961; Rao and Ballard, 1999), where prediction is performed on the basis of statistical properties of sounds, rather than deterministic predictions: these properties are estimated and then compared to incoming data. Consistent with previous work on this hypothesis, we find activation of the change-related potential to derive from the associative cortex and partially from frontal cortex (Wacongne et al., 2011).

## Synthesis

Reviewing Editor: Leonard Maler, University of Ottawa Decisions are customarily a result of the Reviewing Editor and the peer reviewers coming together and discussing their recommendations until a consensus is reached. When revisions are invited, a fact-based synthesis statement explaining their decision and outlining what is needed to prepare a revision will be listed below. Note: If this manuscript was transferred from JNeurosci and a decision was made to accept the manuscript without peer review, a brief statement to this effect will instead be what is listed below.

I have carefully read the two previous reviews, your rebuttal and the

revised manuscript.

I think you have carefully addressed all the fairly minor points raised

by the reviewers. The most important criticism was the lack of novelty

of this Ms given the previous work from your group. You have in part

answered this criticism.

I think that there could still be some improvements made that would

increase the relevance of the results for a wider audience.

In the Introduction, you now provide a very clear hypothesis:

“Our main hypothesis is that change detection in complex,

statistically defined, textural stimuli requires the accumulation of

sensory evidence, and that the degree to which the neural system

performs the accumulation covaries with the level of task involvement.”

Please, in the Discussion, summarize in clear and simple terms the

status of your hypothesis - I believe that you provide convincing

evidence in its favour, but this is not really clearly specified and it

should be. This might go together with the nice ending re the

‘predictive coding framework’.

Your Rebuttal mentions the controversy re the site of evidence

integration; this should be more explicitly noted in the Discussion with

emphasis on your better localization. What I found most interesting was

the O'Connell et al reference since this was very general, i.e., not

confined to a single sense. It would be good to discuss (briefly)

whether you think your results are also generalizable to the visual and

somatosenory systems - this would certainly make this ms 'of general

interest'.

I realize that my requests will take some space in the Discussion; but I

did find that the Discussion overemphasized some fairly minor points.

I think it can be re-written to make it more general without losing too

much detail relevant to specialists in your field.

## Author Response

Response Letter for the eNeuro Manuscript #eN-TNWR-0090-18X

- Extended Data Figure 4-1 needs to be referenced in the legend for Figure 4. R: We have added the corresponding Reference to Fig.4.

- RRIDs:

R: There are no RRIDs relevant to the current manuscript to report.

In the Introduction, you now provide a very clear hypothesis: “Our main hypothesis is that change detection in complex, statistically defined, textural stimuli requires the accumulation of sensory evidence, and that the degree to which the neural system performs the accumulation covaries with the level of task involvement.”

Please, in the Discussion, summarize in clear and simple terms the status of your hypothesis - I believe that you provide convincing evidence in its favour, but this is not really clearly specified and it should be. This might go together with the nice ending the ‘predictive coding framework’.

R: Excellent suggestion. We have grouped the new hypothesis evaluation with the last two paragraphs into a Conclusions and outlook section, including the predictive coding framework. This definitely clarified the structure of the last part of the discussion.

Your Rebuttal mentions the controversy re the site of evidence integration; this should be more explicitly noted in the Discussion with emphasis on your better localization. R: We think this is overall a complicated question, which we try to do better justice now in the revised section “Neural Origin of Change Related Activity”. In comparison to a number of studies from the group of Redmond Kelly, we estimate source rather than interpret scalp level potentials. On the other hand, the localization performed by the group of Maria Chait is likely on par with our techniques, hence, it may rather be task differences that lead to different sources.

What I found most interesting was the O'Connell et al reference since this was very general, i.e., not confined to a single sense. It would be good to discuss (briefly) whether you think your results are also generalizable to the visual and somatosensory systems - this would certainly make this ms ‘of general interest’.

R: Agreed, the O'Connell et al. Nat Neuro. 2012 is really an excellent paper with a number of important controls, including the supramodality. We included a consideration of the question of generalization to other systems in the discussion, in the section ‘Neural responses across different levels of task-engagement’, since the translation to other modalities is most relevant here, in our opinion.

I realize that my requests will take some space in the Discussion; but I did find that the Discussion overemphasized some fairly minor points. I think it can be rewritten to make it more general without losing too much detail relevant to specialists in your field. R: We have carefully gone through the discussion, shortening/removing a few wieldy sections, which allowed us to slightly shorten the discussion, despite the additions requested (1837 before vs. 1799 now). In particular we deleted one lengthy subsection in the heading ‘Relation to previous work on change detection in complex acoustic changes’, which was already addressed in the paragraph on the origin of the signals.
