# Supplementary figures and images for: Evidence Integration in Natural Acoustic Textures during Active and Passive Listening
Source: eNeuro. 2018 Apr 13;5(2):ENEURO.0090-18.2018. doi: 10.1523/ENEURO.0090-18.2018 (PMC5898696; doi:10.1523/ENEURO.0090-18.2018)

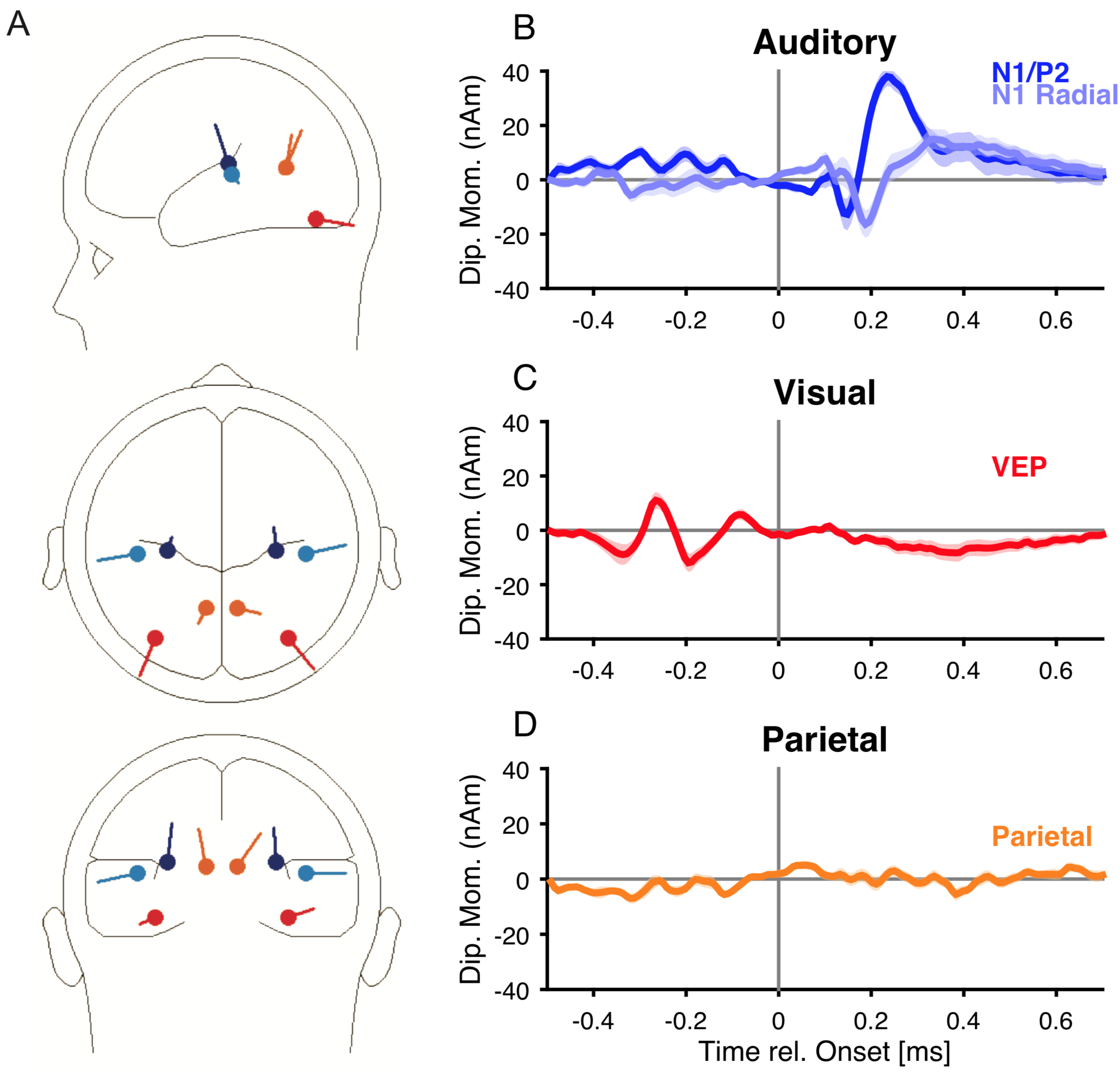

Supplement: Extended Data Figure 3-1 — The parietal potential at and before stimulus onset has a visual origin (A, C). The auditory response can be well explained by two orthogonal dipole sources located in the auditory cortex (A, B). The parietal dipole (D) remains quiet at the onset of the stimulus, indicating that the negative potential around 240 ms can indeed be accounted for by a mixture with the auditory cortex source(s) (see B). The residual variance of this estimate was 1.48%. Download Figure 3-1, TIF file. [file sup_enu-eN-NWR-0090-18-s03.tif]

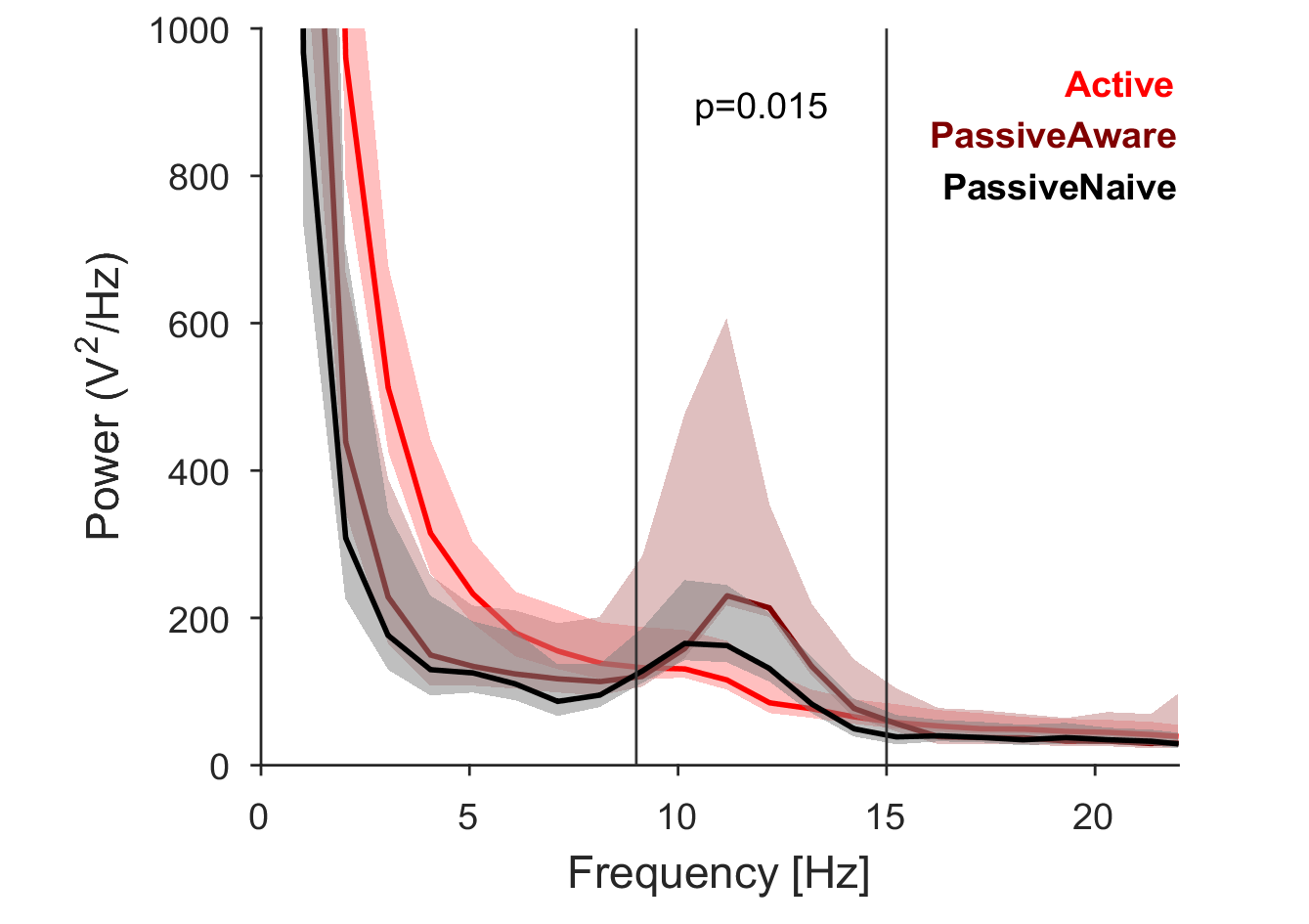

Supplement: Extended Data Figure 3-2 — Distribution of spectral power in different levels of task involvement. Active subjects show barely elevated power in the α-band (red, n = 18). The passive-aware subjects showed elevated α-band activity around 10–15 Hz (maroon, n = 8). The passive-naive subjects showed elevated α-band activity around 9–14 Hz (black, n = 10). Spectra were computed from channel Oz. The difference between the three conditions was significant (p < 0.001, two-way ANOVA, on frequency (df = 5, range 9–15 Hz, vertical lines) and condition, df = 2). The elevated α-band can be taken as an indication for a reduced level of task engagement in both passive groups compared to the active group. The power spectra were computed in the 1.5 s preceding the start of the stimulus to avoid contributions of the task-related ERPs. Outside the depicted frequency range, the spectra were quite similar. Error hulls represent ±1 SEM. Download Figure 3-2, TIF file. [file sup_enu-eN-NWR-0090-18-s04.tif]

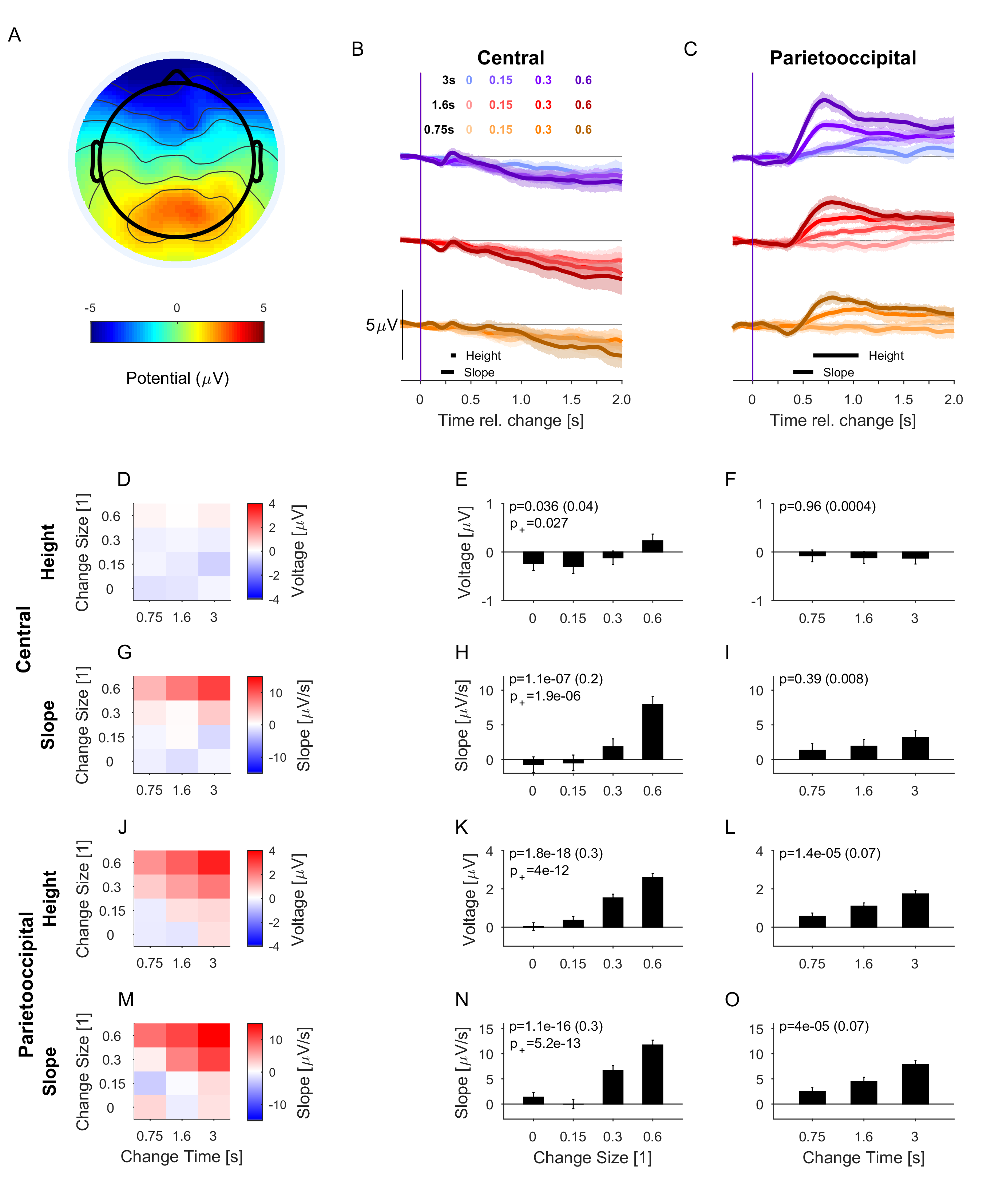

Supplement: Extended Data Figure 4-1 — Same analysis as in Figure 4 but for all trials. Nomenclature identical to Figure 4. Demonstrates that the variability was very similar across conditions; however, the number of trials per condition differed across hits and misses. Note that the strong dependence of slope and height on change size and change height is due to the larger number of miss trials for small change sizes and change times. Download Figure 4-1, TIF file. [file sup_enu-eN-NWR-0090-18-s01.tif]
